# Deicing salt exacerbates freshwater salinization under climate change and human activities

**DOI:** 10.1016/j.xinn.2025.100862

**Published:** 2025-02-26

**Authors:** Ziyong Zhao, Wenyu Yang, Pei Hua, Peter Krebs, Jin Zhang

**Affiliations:** 1 Institute for Ecological Research and Pollution Control of Plateau Lakes, Yunnan University, Kunming 650500, China; 2 School of Geography, South China Normal University, Guangzhou 510631, China; 3 Institute of Urban and Industrial Water Management, Technische Universität Dresden, 01062 Dresden, Germany; 4 Chair of Engineering Hydrology and Water Management, Technische Universität Darmstadt, 64287 Darmstadt, Germany; 5 Department of Hydrogeology, Helmholtz Centre for Environmental Research – UFZ, 04318 Leipzig, Germany; 6 Guangdong Provincial Key Laboratory of Chemical Pollution and Environmental Safety, School of Environment, South China Normal University, Guangzhou 510006, China; 7 The National Key Laboratory of Water Disaster Prevention, Yangtze Institute for Conservation and Development, Hohai University, Nanjing 210098, China

## Main text

Extreme rainfall events caused by climate change are triggering the instability of water resources, while the expansion of human activities is contributing to water quality degradation, jointly intensifying the risk of water scarcity. It has been reported that 40% of the world’s population is at risk of quantity-based water scarcity for at least 1 month a year, while 26% are at risk of quality-based water scarcity.^1^ Increasing human activity is projected to exacerbate water quality degradation in the coming years, which poses a threat to global water security. These issues are closely related to the United Nations Sustainable Development Goals (SDGs), including human health and well-being (SDG 3), ensuring access to water resources and sanitation (SDG 6), and promoting sustainable urban and community development (SDG 11).^2^ Therefore, it is necessary to develop an integrated management response to these issues.

Among these threats, freshwater salinization poses a significant challenge to quality-based water scarcity. The increasing salinity of inland freshwater globally, termed "freshwater salinization syndrome," threatens both water quality and aquatic ecosystems. Previous studies have focused on natural drivers—such as climate change, seawater intrusion in coastal areas, and geological salinization in inland regions—as well as anthropogenic factors like irrigation backflow, chemical pollution, and urbanization. Recently, the role of deicing salts in freshwater salinization has received attention. The majority of deicing salts are chloride-based compounds (e.g., sodium chloride [NaCl], calcium chloride [CaCl_2_], magnesium chloride [MgCl_2_], and potassium chloride [KCl]), which lower the melting point of snow and ice to ensure road safety. For instance, the common application rate of road salt in the northeastern United States is 84 kg/km (300 lb/mile), and multiple snowfall events result in an annual application rate of 15–30 tons per mile (8.45–16.9 kg/m).^3^ As the snow melts, large quantities of salts are transported into nearby rivers, lakes, and groundwater, thereby increasing freshwater salinity. However, due to limited data availability, the drivers and environmental impacts of deicing salt remain unclear, posing challenges for managing lake salinization. It is crucial to reveal the migration mechanism of deicing salts—considering potential drivers such as climate patterns, population growth, and urban expansion—and characterize their temporal trends and spatial distribution under changing environments. These research gaps present significant challenges for developing and implementing effective response strategies, especially given the current imbalance in freshwater resources.

## Salinization due to deicing salt threatens the environment and society

Deicing-salt-induced salinization has been proven to have a series of negative environmental and social impacts. With rainfall or snowmelt runoff during early spring and winter, large amounts of chloride ions from deicing salts are carried into nearby receiving waters, delivering a significant shock to the local water environment. Several studies indicate that once the salinity of surface water reaches a certain threshold, the ecosystem may undergo a non-linear and abrupt change, leading to ecosystem collapse, significant disruptions to food webs and ecological processes, or reduced biodiversity as sensitive species are replaced by more salt-tolerant organisms.^4^ This threshold may vary spatially and temporally with changing climate and intensified human activities, further complicating freshwater salinization management. Concurrently, as snow containing deicing salts melts and infiltrates the soil, substantial chloride remains, resulting in soil salinization and gradual release into groundwater during subsequent precipitation events. This process could lead to the long-term deterioration of groundwater quality, which would be detrimental to achieving SDG 6 on water resources and sanitation.

The negative impacts of deicing salts extend beyond the environment. The World Health Organization (WHO) has identified a correlation between contaminated water, poor sanitation, and the transmission of diseases. Long-term excessive intake of chloride ions may burden the kidneys and increase the risk of hypertension, while high chloride concentrations in water can corrode distribution infrastructure (e.g., pipes and valves), leading to increased maintenance and replacement costs. Such costs may be borne by residents and businesses, thereby discouraging sustainable urban and community development (SDG 11). However, due to insufficient data—such as reliable figures on deicing salt usage and key migration parameters—the environmental and social risks associated with deicing salts remain assessed largely on a qualitative basis. For instance, the absence of reliable data on deicing salt usage makes it challenging to assess the effectiveness and environmental implications of different regional application strategies. Similarly, the limited understanding of key parameters governing the migration of deicing salts hinders the quantification of their flux and ecological impact across terrestrial and aquatic ecosystems. Moreover, insufficient information about long-term health effects restricts the ability to evaluate potential risks to public health.

## Multiple factors contribute to deicing salt pollution

**Figure 1:**
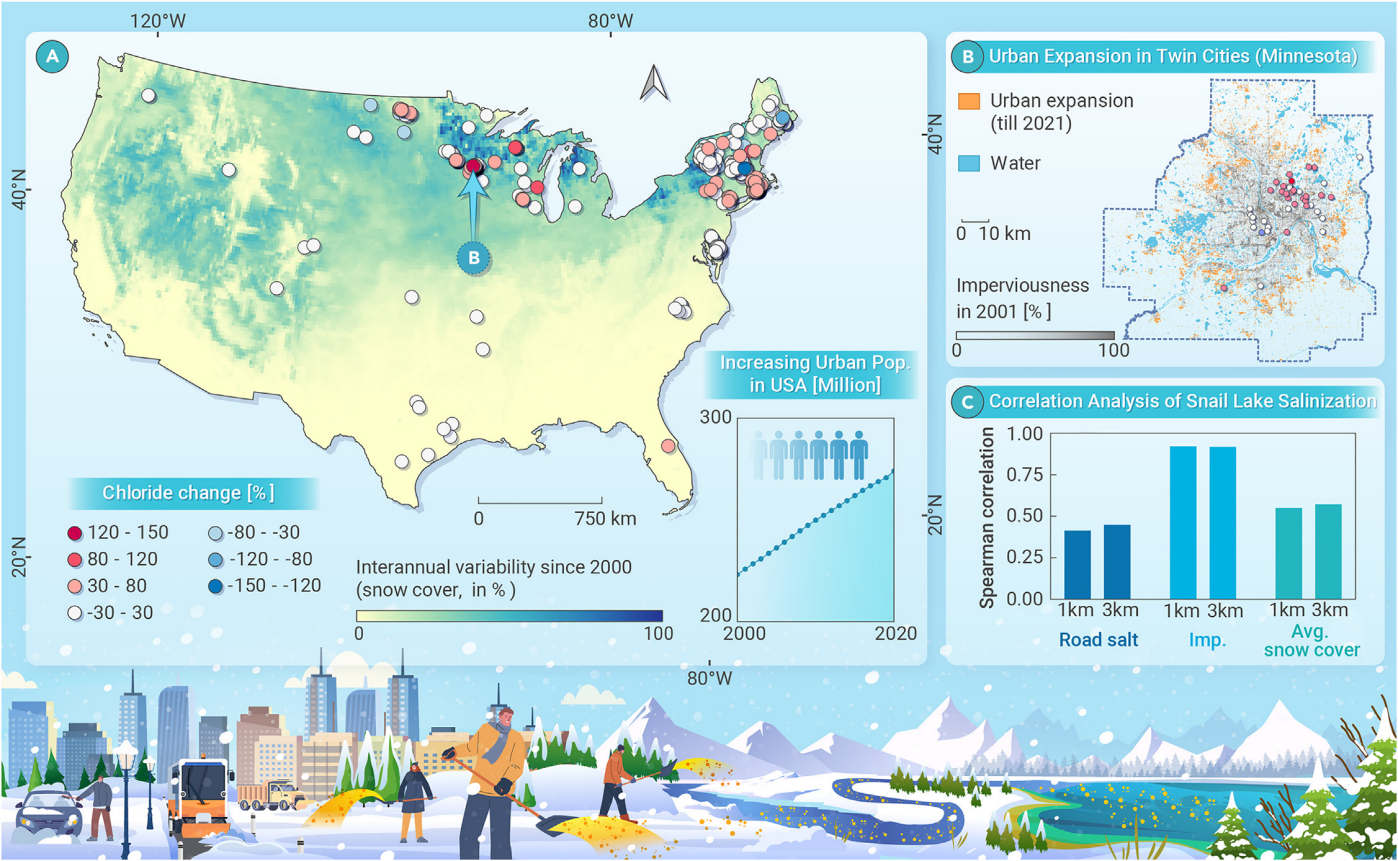
The impact of deicing salt on freshwater salinization (A) Interannual variability of snow cover across the United States, long-term chloride trends in freshwater lakes, and urban population growth over the past two decades. (B) Urban expansion in the Twin Cities (Minnesota) and long-term chloride trends in local freshwater lakes. (C) Correlation analysis of Snail Lake salinization.

The impact of climate change and human activities has significantly increased the complexity of deicing salt pollution. For example, Figure 1A demonstrates that the interannual variability of seasonal snow cover in the northeastern United States and around the Great Lakes has exceeded 50% over the past 20 years, making it difficult to plan deicing salt usage based on historical data alone. Long-term monitoring data (Figures 1A and 1B) also show a clear trend of lake salinization, underscoring the urgency of addressing this issue.

The contribution of human activities should not be overlooked. In the United States, the urban population has increased by about 50 million over the past 20 years (Figure 1A), and accelerated urbanization has led to an increased demand for roads and infrastructure, resulting in a notable surge of deicing salt during snow seasons. Taking the Twin Cities of Minnesota as an example, the area of impervious surfaces has increased by about 400 km^2^ in the past 20 years, and freshwater lakes in the northeast of this region face severe salinization problems. The chloride change in Lake Snail, one of the most serious cases, has increased by more than 100%. Correlation analysis (Figure 1C) indicates that deicing salt consumption, surface imperviousness, and snow cover were positively correlated with lake salinization, which provides insights into understanding the mechanism of freshwater salinization. Further research is, therefore, necessary to quantify the response of lake salinity to these factors.

## Addressing deicing salt pollution in a changing environment

Effectively mitigating the environmental and social impacts of deicing salt contamination requires a multifaceted approach that integrates technical solutions with targeted measures. Firstly, it is important to characterize the migration and distribution of deicing salts using a combination of field sampling, chemical assays, and geographic regression analyses. Hydrological and hydrodynamic models can clarify the spatial-temporal migration characteristics of deicing salts during snowmelt or rainfall events, although challenges in complexity and accuracy remain. In this regard, machine learning techniques offer additional support for handling large datasets, improving prediction accuracy, and identifying complex patterns. Quantitative statistical analyses can help visualize the pollution risk in receiving waters, thereby providing a scientific basis for deicing salt control strategies.

Controlling salt usage at the source is among the most effective strategies to prevent pollution. In urban areas with sufficient infrastructure or regions with high environmental sensitivity, alternative snow removal measures, such as mechanical snow removal or transferring snow to designated storage areas, can be adopted. For instance, in Harbin (northern China), policies have been introduced to strictly limit deicing salt usage and prioritize mechanical snow removal to minimize environmental impacts. Furthermore, the use of environmentally friendly alternatives (e.g., organic and biological salts) is encouraged. Although these alternatives may be more expensive than traditional deicing salts, they can reduce risks to water ecosystems while maintaining deicing effectiveness. Pilot programs in the United States and Canada have explored organic deicers like beet juice, carbohydrate-enhanced chemicals, and cheese or dairy brines to mitigate the harmful effects of chlorides on freshwater systems. In addition, deicing salt recycling represents a promising area for future research. For instance, the high-salinity snowmelt runoff from cities with separate drainage systems can be treated by membranes to recycle the deicing salt. This approach not only reduces the burden on the environment but also facilitates the reuse of resources. However, the feasibility and economic benefits require further investigation. Nature-based solutions (such as bioretention systems and permeable asphalt) have demonstrated the potential to mitigate pollution caused by deicing salt, provided that the specific challenges of low temperatures and high salinity are adequately addressed.^5^

Finally, the development of robust standards is essential for promoting integrated water resource management. To achieve the core SDG concepts of health, clean water, sustainable cities, and ecosystem protection, countries must strengthen policy support. Policies like China’s “Water Ten Plan,” the United States’s “Clean Water Act,” and the EU’s “Water Framework Directive” serve as examples of how integrating policy, management, and international cooperation can protect the water environment and human health.

## Funding And Acknowledgments

This work was jointly supported by the 10.13039/501100001809National Natural Science Foundation of China (NSFC; grant nos. 42077156 and 42377059) and the 10.13039/501100004543China Scholarship Council (CSC; grant nos. 202008080005 and 202408080217). This work has not been subject to peer review by the above agencies and does not, therefore, reflect the views of the above agencies, nor should any official endorsement be inferred. The funders had no role in the study design, data collection and analysis, decision to publish, or preparation of the manuscript.

## Declaration of interests

The authors report no conflicts of interest.
